# Quantification of Arachidonic Acid and Its Metabolites in Rat Tissues by UHPLC-MS/MS: Application for the Identification of Potential Biomarkers of Benign Prostatic Hyperplasia

**DOI:** 10.1371/journal.pone.0166777

**Published:** 2016-11-28

**Authors:** Qiaoxia Bian, Weihui Wang, Nannan Wang, Yan Peng, Wen Ma, Ronghua Dai

**Affiliations:** School of Pharmacy, Shenyang Pharmaceutical University, Shenyang 110016, China; Boston University, UNITED STATES

## Abstract

To evaluate the potential relationship between benign prostatic hyperplasia (BPH) and the arachidonic acid (AA) metabolome, a UHPLC—MS/MS method has been developed and validated for simultaneous determination of AA and its cyclooxygenase(COX) and lipoxygenase(LOX) pathway metabolites (15-HETE, 12-HETE, TXA_2_, 5-HETE, AA, PGI_2_, PGF_2α_, 8-HETE, PGD_2_, PGE_2_ and LTB_4_) in rat tissues. The analytes were extracted from tissue samples with a protein precipitation procedure and then separated on a Shim-pack XR-ODSC18 column with 0.05% formic acid in water (pH adjusted with dilute ammonia) and methanol:acetonitrile (20:80, v/v). Detection was performed on a UHPLC—MS/MS system with electrospray negative ionization (ESI) and a multiple reaction-monitoring mode. The lower limits of quantification (LLOQ) were 0.25–50 ng/mL for all of the analytes in the prostate, seminal, bladder, liver and kidney tissues. The absolute recoveries of the analytes from all of the tissues were more than 50%. By means of the method developed, the AA metabolites in tissue samples from Sham and BPH group rats were determined. The eleven biomarkers in the BPH group prostate, seminal, bladder, liver and kidney tissues were significantly higher than those of the sham group, indicating that BPH fortified the inducible expression of COX and LOX, as well as increased the production of AA and eicosanoids. The method described here offers a useful tool for the evaluation of complex regulatory eicosanoids responses in vivo.

## Introduction

Benign prostatic hyperplasia (BPH) is the most common benign tumor in men, showing an age-related incidence. BPH represents a pattern of unregulated but non-malignant growth characterized by an increase in prostate epithelial and stromal cells [1]. The exact pathogenesis of BPH is still unclear, but several clinical and experimental observations indicate that inflammation in the prostate gland is closely associated with the development of BPH [2, 3, 4]. Approximately 90% of samples taken during transurethral resection of the BPH prostate present prostatic inflammation in histologic diagnosis [5]. Inflammation mediated through the upregulation of lipid and protein mediators released by inflammatory cells contributes to the tissue injury driving local angiogenesis. The most important inflammatory pathway is the metabolism of arachidonic acid (AA) and it has been associated with the development of BPH. Eicosanoids are generated through the cyclooxygenase (COX) and lipoxygenase (LOX) and cytochrome P450 monooxygenase (CYP450) pathways [6]. AA and eicosanoids can severely disrupt cellular signaling processes. Due to the importance of these mediators, it is necessary to systematically evaluate their changes in a biological matrix.

Previous research shows that both COX-1 and COX-2 are expressed in the prostate gland [7, 8, 9]. Prostaglandins (PGs) arising from the transformation of AA mediated by two different isoforms of COX (COX-1 and COX-2) are important inflammatory mediators. In specific pathologic conditions, AA is a substrate for COX-2, an inducible enzyme that generates thromboxane A_2_ (TXA_2_) then TXA_2_ is converted to its biologically inactive metabolite, TXB_2_. Leukotrienes (LTs) and hydroxyeicosatetraenoic acids (HETEs) are characterized metabolites generated through the LOX metabolic pathway. The enzyme 5-LOX enzyme has been reported to be over-expressed in prostate cancer [10]. LTs attract leukocytes to the site of inflammation, promoting their adhesion to the inflamed and damaged tissue [11]. HETEs stimulate proliferation of prostate muscle cells through a MAPK-dependent mechanism and cause cerebral micro vessel constriction, which possibly has a role in the remodeling process of inflammation. All of these experimental findings suggest that a dual inhibitor of the COX and 5-LOX enzymes might mediate AA metabolism and act as an anti-inflammatory drug [12]. To date, the physiological and biochemical functions of eicosanoid lipids derived from these two metabolic routes have not been widely studied in a BPH model.

A complete overview of a metabolic profile can provide information on the metabolic pathways in which the disruption of bioactive species may be involved in a disease state. AA metabolites generated through the LOX pathway are highly expressed in blood and immune cells, whereas the metabolisms via COX pathway are highly expressed in tissues. This distribution of metabolite suggests that some endogenous biomarkers can only be detected in the local environment and that these different biomarkers are distributed widely in different tissues in the body. Hence, we chose the prostate, seminal, bladder, liver and kidney tissues to research the changes in AA metabolites that are related to BPH, and to gain better insight on inflammatory processes.

This research aims to develop a metabolomics approach to elucidate the mechanism of eicosanoid metabolite production in different tissues types from rats with BPH. We developed a more versatile UHPLC-MS/MS method for the simultaneous analysis a broad spectrum of related eicosanoids in rat tissues and demonstrated its application in the detection of AA metabolism changes in a rat model of BPH [13].

## Experimental

### Chemicals, reagents and materials

Injectable testosterone propionate was purchased from General Pharmaceutical Co., Ltd. (Shanghai, China). 5-hydroxyeicosatetraenoic acid (5-HETE), 8-hydroxyeicosatetraenoic acid (8-HETE), 12-hydroxyeicosatetraenoic acid (12-HETE), 15-hydroxyeicosatetraenoic acid (15-HETE), arachidonic acid (AA), thromboxane-A_2_ (TXA_2_), thromboxane-B_2_ (TXB_2_), prostaglandin D_2_ (PGD_2_), prostaglandin E_2_ (PGE_2_), prostaglandin F_2α_ (PGF_2α_), prostaglandin I_2_ (PGI_2_), leukotriene-B_4_ (LTB_4_), leukotriene-D_4_ (LTD_4_), arachidonic acid-d_8_ (AA-d_8_), 15-hydroxyeicosatetraenoic acid-d_8_ (15-HETE-d_8_), prostaglandin D_2_-d_4_ (PGE_2_-d_4_) and 5-hydroperoxyeicosatetraenoic acid (5-HPETE) were purchased from Cayman Chemicals (Ann Arbor, MI, USA). Deuterated compounds (AA-d_8_, 15-HETE-d_8_, PGE_2_-d_4_) were used as internal standards for quantification. The purities of all of those reference standards were more than 98%. Distilled water was obtained from Wahaha Co., Ltd. (Hangzhou, China). Methanol and acetonitrile of HPLC grade were bought from Fisher Scientific (Fair Lawn, NJ, USA). HPLC grade formic acid and ammonium hydroxide were provided by Shandong Yuwang Industrial Co., Ltd. (Yucheng, China). All of the other reagents were of analytical grade.

### Animals and treatment

Twelve male Sprague-Dawley rats (180–200g) were obtained from the Experimental Animal Center of Shenyang Pharmaceutical University. The rats were allowed to acclimate for 7 days prior to experiments. The animals were kept in an air-conditioned room at a relative humidity of 40–60% and at a temperature of 25 ± 2°C. The rats had free access to the standard laboratory food and water and were housed under a 12-h light/dark cycle. The animal experiment was carried out in accordance with the Guideline for Animal Care and Use Committee and Animal Experimentation of Shenyang Pharmaceutical University, and the protocol was approved by the Animal Ethics Committee of the Institutional Animal Care and Use Committee (IACUC) and Animal Experimentation of Shenyang Pharmaceutical University (SYPU) (NO.SYPU-IACUC-S20150907-02).

The animals were randomly divided into two groups (sham group and BPH model group). After they were anesthetized with chloral hydrate (350 mg/kg), rats in the sham group were cut open and then sewn up without cutting off the testicles, and the rats in the BPH group were castrated. After a 7-day recovery, sham group rats were treated with vehicle (n = 6; 100 μL olive oil, s.c.), and BPH group rats were treated with testosterone propionate (n = 6; testosterone propionate 0.5 mg/kg, s.c. diluted in olive oil in a volume of 100 μL) for 28 days [14]. The body weights were measured once weekly, and the general physical condition of each rat was observed. After 28 days, all of the rats were killed by decapitation, and the prostate, seminal, bladder, liver and kidney tissues were rapidly removed and weighed. The wet weight index was obtained. Prostate ventral lobes and dorsal lobes were fixed in 10% neutral buffered formalin and were embedded in paraffin for histopathological research. The samples were kept frozen at −80°C until use. The tissues were homogenized with three times the volume of acetonitrile with hand-held pestles in a vial and were then vortex mixed for 5 min and centrifuged for 5 min at 13000 rpm. Then, the supernatants were transferred to a vial and were used as the samples.

#### Sample preparation

To an aliquot of 200 μL of prostate sample or blank prostate sample, 20 μL IS [AA-d8 (1.00μg/mL), PGE2-d4 (200.0ng/mL), and 15-HETE-d8 (100.0ng/mL)] and 20 μL methanol:water (50:50,v/v) were added and vortex mixed for 1 min and then the mixture was extracted with 1.0 mL of acetonitrile (containing 0.05% formic acid) by vortex-mixing for 3 min. After centrifugation for 10 min at 13000 rpm (4°C), a 5μL aliquot of the supernatant was injected into the UPLC-MS/MS system for analysis.

### Instruments and analytical conditions

Liquid chromatography separation was performed on an XR LC-20AD ProminenceTM UPLC system equipped with a binary pump, a degasser, an autosampler and a thermostatted column compartment (Shimadzu, Japan). The chromatographic separation of the analytes and IS was performed on a Shim-pack XR-ODS C_18_ column (3.0mm×75mm, 2.2μm particle size, Shimadzu, Japan) protected by a high-pressure column pre-filter (4.0×10mm, 2μm) (Waters, USA) with the column temperature set at 30°C. Chromatographic separation was achieved with gradient elution using a mobile phase comprised of 0.05% formic acid in water, pH = 3.3 adjusted with dilute ammonium hydroxide and acetonitrile:methanol (80:20, v/v) (B). The UHPLC gradient program was as follows: 45% B→80% B at 0.01–3.00 min; 80% B→100%B at 3.01–5.00 min; 100% B at 5.01–6.00 min; 100% B→45%B at 6.01–9.00 min; and 45% B at 9.01–12.00 min. Efficient and symmetrical peaks were obtained at a flow rate of 0.3 mL/min. The sample injection volume was 5μL.

Mass spectrometric detection was carried out on a QTRAPTM 4000 MS/MS system from AB Sciex that was equipped with an electro spray ionization (ESI) source. All of the operations, the acquisition and analysis of data were controlled by Analyst (version 1.5.2, AB Sciex, USA). The detection of the analytes was in the multiple reaction monitoring (MRM) mode using an electrospray negative ionization (ESI^-^) with nitrogen as gas 1, gas 2, and curtain gas set at 50, 40, and 20 psi. The source temperature and ion spray (IS) voltage were set at 500°C and 5500 V, respectively. Multiple reaction monitoring (MRM) measurements of the analytes were performed using declustering potential (DP), entrance potential (EP), collision energy (CE) and cell exit potential (CXP) values optimized for each analyte. The quantitative parameters are listed in S1 Table.

### Method validation

The method was fully validated in the light of the US-FDA document and other relevant guidelines [15, 16]. Calibration standards and QC samples were prepared by adding a dilution of the stock analyte solution to the blank prostate sample on every validation day. The concentrations of analytes in the QC samples were calculated by using calibration curves prepared the same day. All of the standards and QC samples were prepared by the same matrix, which ensures that accuracy, precision, selectivity and sensitivity would not be affected. The samples were extracted as described in the section describing sample preparation and were analyzed by UHPLC—MS/MS.

The specificity was assessed by comparing chromatograms of the blank prostate sample, the blank prostate sample spiked with analytes and IS and prostate sample.

The linearity was generated by plotting the peak area ratio (y) of each analyte subtracted those of blank samples to IS versus nominal concentrations (x) of eicosanoid by 1/x^2^ weighted least square linear regressions.

The lower limits of quantification (LLOQ) were defined as the lowest concentration measurable with precision less than 20% and accuracy within ± 20% deviation.

Accuracy and precision were determined by quantifying three QC levels on the same day (intra-day) and three consecutive validation days (inter-day) with six replicates at each concentration. Precision was expressed as the relative standard deviation (RSD, %) and accuracy was expressed as relative error (RE, %).

The extraction recoveries of the eleven analytes and IS were assessed calculating the ratios of the analyte standard peak areas obtained from extracted samples to that of post-extracted samples including the analytes. Rats higher than 50% can be accepted.

The matrix effects were expressed as the ratios of the peak areas of analytes spiked post-extraction to that analytes dissolved in initial mobile phase at corresponding concentrations.

The stability of the eleven analytes was evaluated using triplicates of spiked samples at three QC levels under different conditions as follows: 4 h at room temperature, three freeze—thaw cycles, stored at −80°C for a month, and reconstituted extract at 4°C for 12 h.

#### Method application

Integrated raw mass spectrometric data were processed using Analyst software (version 1.5.2, AB Sciex, USA). The intensity of each ion was normalized with respect to the total ion count to generate a data matrix consisting of the retention time, the m/z value, and the normalized peak area. The processed data were exported and further processed by PCA and PLS-DA using the SIMCA-P software package (Version11, Umetrics AB, Ume, Sweden). The data were processed by unit variance scaling and were mean-centered using the SIMCA-P software. Potential markers of interest were extracted from the values of variable importance in the projections (VIP>1), which were constructed from PLS-DA analysis. P values were obtained from Student’s T test (P <0.05).

### Data analysis

Values were presented as the means ± standard deviation (SD). The results were analyzed statistically by independent-samples T test using SPSS 16.0 software (Statistical Package for the Social Science) for Windows. Differences were considered statistically significant at P<0.05.

## Results and Discussion

### Optimization of chromatographic and mass conditions

**Fig 1:**
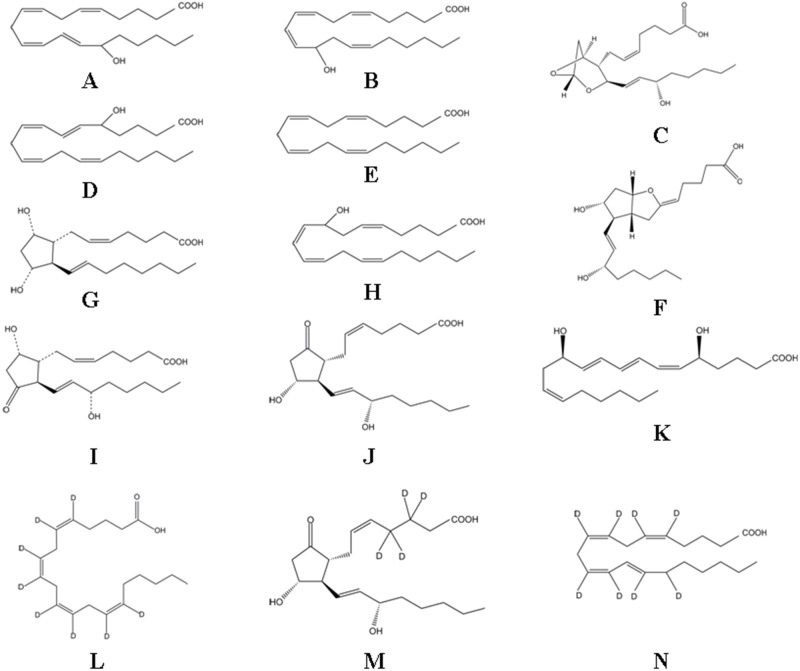
Chemical structures of AA and its metabolites. (A) 15-HETE, (B) 12-HETE, (C) TXA_2_, (D) 5-HETE, (E) AA, (F) PGI_2_, (G) PGF_2α_, (H) 8-HETE, (I) PGD_2_, (J) PGE_2_, (K) LTB_4_, (L) AA-d_8_, (M) PGE2-d_4_ and (N) 15-HETE-d_8_.

The structures of eicosanoids are presented in Fig 1 [17]. As most metabolites in the AA metabolome are COOH^-^ containing lipids with highly charged anions, ESI^−^ mode was employed for the analysis. Cone voltage and collision energy parameters were optimized. The other parameters followed the recommended value of the instrument. Methanol:acetonitrile (20:80, v/v) was used as the organic modifier because of its higher sensitivity and lower background noise than single methanol or acetonitrile, and the with addition of formic acid and ammonium to the mobile phase, the sensitivity and peak symmetry of AA metabolome and IS were clearly improved. Furthermore, the concentration of formic acid from 0.01% to 0.2% was optimized and the different buffers of varying pH were tested. The results indicate that the addition of 0.05% formic acid and ammonium to pH 3 into the water phase worked best. Because it dramatically enhanced the response of eicosanoids with an adequate LLOQ, and it slightly suppressed the intensity of the eicosanoids. Finally, the optimized gradient elution mentioned in ‘Instrumentations and analytical conditions’ was adopted to separate the analytes.

### Sample preparation

Given the complexity and variety of the matrices, several clean-up strategies were evaluated, such as protein precipitation, liquid—liquid extraction, and solid-phase extraction. Owing to the high-polarity of all of the analytes and IS, adding five volumes of acetonitrile to induce protein precipitation showed a satisfactory recovery for all of the analytes. In the meantime, the tissue sample preparation parameters including volume of extraction solvent, centrifugation time and centrifugation rotation speed also need to be optimized.

### Method validation

#### Specificity

**Fig 2:**
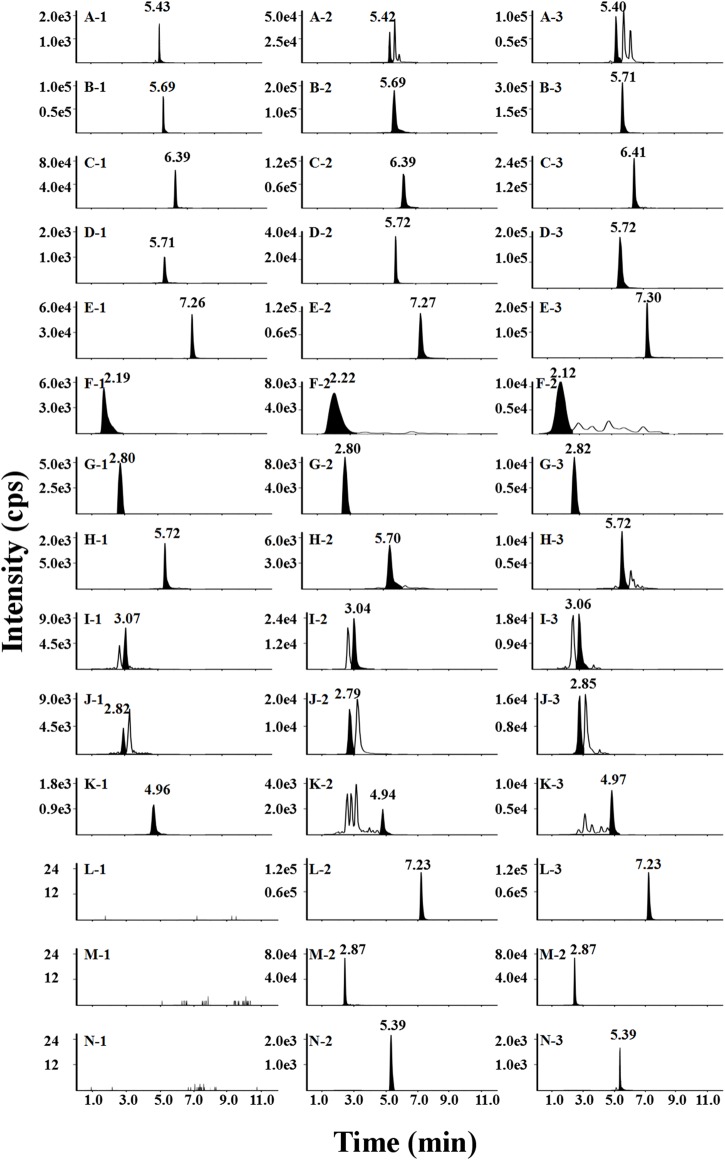
Representative chromatograms of AA and its metabolites in blank prostate sample (1), blank prostate sample spiked with the analytes (2), A to N at LLOQ and a prostate sample from a BPH rat (3). (A) 15-HETE, (B) 12-HETE, (C) TXA_2_, (D) 5-HETE, (E) AA, (F) PGI_2_, (G) PGF_2α_, (H) 8-HETE, (I) PGD_2_, (J) PGE_2_, (K) LTB_4_, (L) AA-d_8_, (M) PGE_2_-d_4_ and (N) 15-HETE-d_8_

The specificity of the method was established with a blank prostate sample, a blank prostate sample spiked with the analytes and IS at the LLOQ and a prostate sample from BPH rats. The results showed no interference with analytes and IS, as shown in Fig 2.

#### Linearity and LLOQ

The calibration curve of the assay was generated by analyzing samples with a mixture of eleven calibration standards over the concentration range (50–20000 ng/mL for AA; 0.625–250 ng/mL for TXA_2_ and 8-HETE; 1.25–500 ng/mL for 15-HETE, PGD_2_, 5-HETE, PGF_2α_ and LTB_4_; 1–400 ng/mL for PGE_2_ and PGI_2_; 0.25–100 ng/mL for 12-HETE). All the correlation coefficients were greater than 0.9943, exhibiting good linearity. Regression equations, correlation coefficients and linear ranges for the eleven analytes in the prostate are summarized in S2 Table. The LLOQ (50 ng/mL for AA; 0.625 ng/mL for TXA_2_ and 8-HETE; 1.25 ng/mL for 15-HETE, PGD_2_, 5-HETE, PGF_2α_ and LTB_4_; 1 ng/mL for PGE_2_ and PGI_2_; 0.25 ng/mL for 12-HETE) of eleven analytes in prostate are also listed in Table 1. The results above suggested that this method was successful and sensitive enough for the simultaneous determination of the arachidonic acid metabolome.

**Table 1:** Summary of accuracy, precision, recovery and matrix effect of the eleven analytes in rat prostate (n = 6).

Analyte	Concentration (ng/ml)	Intra-day RSD (%)	Inter-day RSD (%)	Accuracy (RE %)	Recovery (%, mean ± SD)	Matrix effect (%, mean ± SD)
15-HETE	2.5	6.1	4.1	1.5	94.3 ± 8.4	86.3 ± 2.7
	25	6.5	1.9	−7.2	93.4 ± 1.9	109.9 ± 4.0
	400	1.5	8.1	3.9	97.6 ± 3.9	99.6 ± 4.7
12-HETE	0.5	7.5	4.8	1.4	96.7 ± 2.8	91.6 ± 4.2
	5	4.9	0.8	−5.4	74.8 ± 3.1	89.3 ± 6.6
	80	9.2	7.2	−3.5	77.7 ± 3.4	86.2 ± 3.1
TXA_2_	1.25	9.5	5.3	−2.9	94.4 ± 4.5	105.7 ± 6.9
	12.5	6.2	8.6	4.7	95.5 ± 9.3	88.1 ± 7.4
	200	4.6	3.5	−0.8	84.3 ± 6.2	86.2 ± 5.3
5-HETE	1	6.0	8.3	2.5	79.4 ± 0.9	100.4 ± 0.9
	10	2.9	9.8	2.5	89.6 ± 8.4	106.8 ± 5.8
	160	8.3	8.9	2.0	73.7 ± 8.6	101.5 ± 0.3
AA	1 00	6.0	8.5	−4.4	72.5 ± 7.0	93.6 ± 6.7
	10 00	9.8	6.8	−0.7	92.2 ± 5.4	99.1 ± 7.3
	1 6 000	3.6	4.5	0.5	81.2 ± 5.3	107.9 ± 2.8
PGI_2_	2	1.9	10.1	−3.7	93.9 ± 2.0	104.9 ± 4.9
	20	0.5	4.5	0.3	84.7 ± 3.1	86.4 ± 7.1
	320	1.9	2.1	0.4	78.3 ± 3.7	95.7 ± 2.5
PGF_2α_	2.5	5.8	6.7	−4.0	98.6 ± 6.9	107.8 ± 9.4
	25	6.5	6.1	−3.5	87.8 ± 7.9	86.9 ± 8.4
	400	9.1	2.1	1.0	91.3 ± 6.9	104.7 ± 3.3
8-HETE	1.25	5.0	1.3	6.1	88.0 ± 6.4	90.6 ± 8.8
	12.5	4.0	4.3	−0.1	90.7 ± 5.8	105.2 ± 9.6
	200	9.6	9.2	−0.8	92.9 ± 6.6	94.9 ± 7.2
PGD_2_	2.5	2.7	3.5	0.1	98.5 ± 4.5	107.9 ± 7.4
	25	9.0	8.3	4.4	82.4 ± 8.4	85.9 ± 6.2
	200	8.5	8.6	0.6	97.5 ± 4.0	102.1 ± 9.6
PGE_2_	2	2.0	2.5	−3.9	75.7 ± 2.2	85.7 ± 9.0
	20	9.3	9.7	−4.6	94.8 ± 2.3	92.1 ± 2.3
	200	5.3	9.4	3.2	72.1 ± 6.4	105.7 ± 1.8
LTB_4_	2.5	9.0	3.8	−8.5	84.4 ± 9.1	100.9 ± 8.8
	25	7.1	2.9	4.0	92.4 ± 7.2	100.5 ± 6.8
	400	5.2	2.1	−0.8	94.8 ± 7.6	93.0 ± 5.1

#### Precision and accuracy

Table 2 summarizes the results obtained for the intra-day and inter-day precision and accuracy of the analytes. The variations in intra-day and inter-day precision were not more than 9.8% and 10.1%, respectively. Accuracy for the eleven analytes was within ±8.5% in prostate tissue samples.

**Table 2:** Stability of the eleven analytes in rat plasma under different storage conditions (n = 3).

Analyte	Concentration (ng/ml)	Room temperature for 12 h		Autosampler vials for 12 h		Three freeze-thaw cycles		−80°C for 14 days	
		RE (%)	RSD (%)	RE (%)	RSD (%)	RE (%)	RSD (%)	RE (%)	RSD (%)
15-HETE	2.5	4.4	8.2	−2.1	6.0	−3.5	6.1	1.5	4.1
	25	−4.8	9.1	−8.1	3.0	−1.3	2.3	−2.2	4.8
	400	−0.9	3.4	4.5	10.9	−5.0	11.3	0.3	5.5
12-HETE	0.5	−5.9	3.6	3.0	12.7	0.1	7.5	1.4	4.8
	5	3.8	6.5	−4.6	11.6	4.0	9.1	0.5	14.1
	80	4.1	8.1	1.4	10.8	−7.8	14.7	0.4	7.4
TXA_2_	1.25	−3.0	4.2	6.2	14.3	5.0	10.5	−2.9	5.3
	12.5	2.9	7.9	2.3	7.7	−0.6	3.7	−5.0	8.3
	200	−0.1	0.7	−2.2	7.3	−1.0	3.5	−4.1	6.9
5-HETE	1	6.2	10.7	7.4	5.5	4.0	11.0	2.5	8.3
	10	2.8	5.5	3.1	12.0	0.6	1.8	1.8	5.3
	160	−5.9	10.3	5.2	11.5	−7.4	12.0	4.8	1.9
AA	1 00	−4.1	8.8	2.0	4.4	3.0	6.0	4.1	2.6
	10 00	0.3	3.9	−2.3	4.3	5.4	11.1	−3.7	11.1
	1 6 000	−1.9	3.8	1.0	2.0	0.5	5.6	−4.1	14.5
PGI_2_	2	1.4	8.3	5.6	10.5	4.1	11.9	3.0	6.5
	20	2.2	9.9	−4.2	6.9	4.2	4.3	−4.0	6.7
	320	3.1	5.6	1.1	2.6	−4	0.9	−2.0	6.6
PGF_2α_	2.5	−3.3	8.8	3.8	6.9	2.3	5.8	5.3	14.0
	25	−6.1	10.5	−3.0	5.1	−0.2	1.3	6.1	11.3
	400	−0.3	9.0	4.7	11.8	−6.6	11.6	−1.0	10.4
8-HETE	1.25	1.1	2.8	−3.5	9.5	3.0	5.0	2.8	6.5
	12.5	1.0	2.1	−0.3	3.9	−0.5	6.7	−10.5	16.9
	200	2.1	5.9	3.2	6.5	−5.4	12.8	−1.9	8.3
PGD_2_	2.5	8.7	6.5	4.7	13.8	−6.9	12.7	3.1	12.1
	25	2.2	9.5	1.6	6.9	1.4	9.1	−4.1	8.8
	200	−2.7	10.8	7.3	4.5	−7.0	14.2	−4.0	5.3
PGE_2_	2	−0.7	3.9	7.4	13.8	4.6	12.0	−3.9	12.5
	20	−3.5	10.2	−5.1	11.1	0.1	7.8	1.5	9.9
	200	1.1	2.8	3.0	12.5	−3.2	6.4	−2.5	7.0
LTB_4_	2.5	−1.1	6.2	5.9	11.0	4.0	9.0	−8.5	3.8
	25	5.4	1.9	0.9	2.0	0.1	5.4	0.5	1.3
	400	4.8	9.0	−3.1	5.3	0.5	0.6	−4.8	8.9

#### Recovery and matrix effect

The recoveries of all eleven analytes exceeded 50% at different concentration levels from prostate tissue samples (Table 2), and the extraction recoveries of IS (AA-d_8_, PGE_2_-d_4_, 15-HETE-d_8_) were 79.1±6.7%, 85.4±1.8% and 80.1±5.7% (mean ± SD) respectively, which proved the extraction process was stable and effective. Matrix effects of the eleven analytes ranged from 85.7 to 107.9% at the three QC levels in prostate tissue samples (Table 2) and the matrix effect of IS was 95.9±2.7%, 89.6±5.6%, and 85.5±1.1% (mean ± SD), indicated that no significant matrix effect was observed for all of the analytes in the prostate samples.

#### Stability

All results for the stability samples tested are summarized in Table 2. The analytes were stable within a 15% standard deviation. In conclusion, the developed analytical method was validated for the accurate and precise measurement of eicosanoids from rat prostate tissue samples. Additionally, this method can be applied to other bio-samples with minor modifications.

### Basic physical parameters

Prostate weight and prostate index (PI) are two important factors to evaluate the development of BPH. Prostate weight (mg) in sham rats and testosterone-treated rats were 606.18±86.40 and 1532.93±12.17 (mean ± SD, n = 6), respectively, and prostate index ×10^−3^ were 1.94±0.23 and 5.35±0.39 (mean ± SD, n = 6), respectively. The data suggest that the BPH model group showed a significant increase in prostate weight and prostate index by 2.6 fold compared to the sham group. These phenomena proved the success of the model.

### Histological analysis

**Fig 3:**
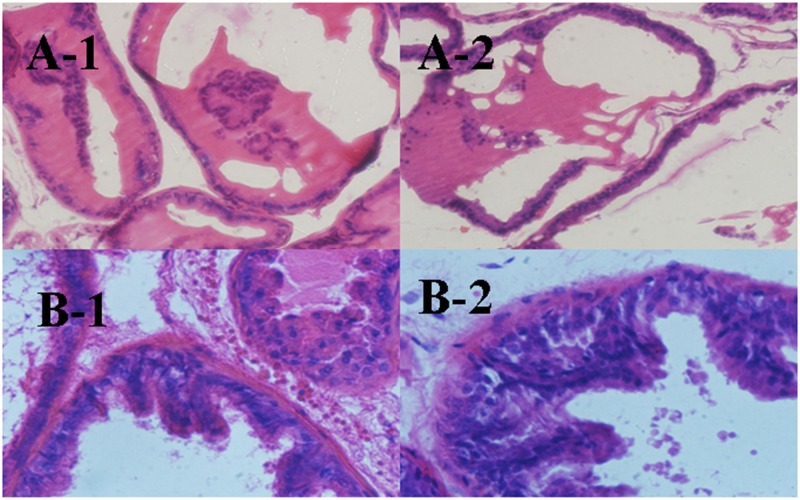
Hematoxylin and eosin staining of prostate ventral lobe (1) and dorsal lobe (2) from sham group rats (A) and BPH induced rats (B).

Prostates were routinely processed and embedded in paraffin, and 5 mm-thick sections were cut and stained with hematoxylin—eosin. The histological architecture of the prostate gland was shown clearly in Fig 3. Prostate glands harvested from sham group rats showed regular acini with cuboidal and low cylindrical epithelium with round nuclei of basal alignment. Fine stroma and a continuous basal layer are also noted (Fig 3A). By contrast, prostates obtained from BPH group rats treated with testosterone propionate had an irregular acinar shape with papillary projection into the lumen and foci of piling-up hyperplastic nodules are evident. The epithelium is highly cylindrical and multilayered, and round/ovoid nuclei are irregularly aligned (Fig 3B).

### Inflammatory factor changes in five tissues

**Fig 4:**
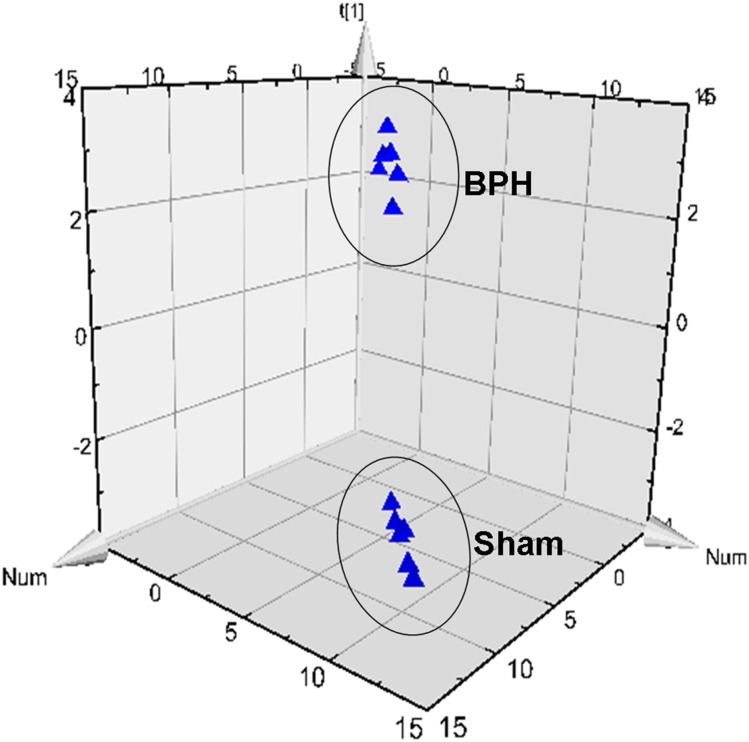
Results of multiple pattern recognition of prostate biomarkers between sham group (sham) and BPH group (BPH). PLS-DA score plot (R^2^*x* = 0.868, R^2^*y* = 0.989, Q_2_ = 0.985, n = 6) of sham and BPH.

The method was used to generate a profile of a range of bioactive lipids in different tissues from rats, including prostate, bladder, seminal, liver, and kidney. After PLS-DA processing, the BPH rats were clearly separated from the sham rats, and Fig 4 showed the results of multiple pattern recognition of prostate biomarkers. Variables were also generated based on the values of variable importance in the projection (VIP>1). The concentration of eicosanoid in different tissues from BPH rats and sham rats were shown in Table 3.

**Table 3:** Levels of individual analytes measured in prostate, seminal, bladder, liver and kidney in saline and testosterone-induced rats.

Analyte	prostate		seminal		bladder		liver		kidney	
	Sham	BPH	Sham	BPH	Sham	BPH	Sham	BPH	Sham	BPH
15-HETE	40.5±2.6	227.5±2.9	170.2±22.0	321.6±11.7	234.1±2.9	313.7±1.5	257.9±17.0	506.7±12.5	108.7±3.4	156.8±9.9
12-HETE	7.2±1.8	26.5±2.7	23.6±2.2	43.1±4.6	14.4±5.5	63.0±8.7	32.0±5.2	74.8±13.5	24.0±8.4	31.5±8.7
TXA_2_	ND	BLOQ	0.9±1.4	9.6±0.9	BLOQ	2.7±1.3	8.5±2.5	50.5±4.1	3.8±0.8	27.2±5.8
5-HETE	10.3±2.2	50.8±3.1	12.5±2.6	43.2±7.2	4.1±1.2	40.6±1.7	8.4±2.2	127.6±0.9	15.3±2.7	20.3±14.5
AA	10.0±0.2	14.5±4.3	3.5±0.8	7.2±0.7	1.4±0.6	10.3±2.5	3.5±1.2	8.2±2.0	1.7±1.3	5.1±2.5
PGI_2_	49.3±0.1	337.5±2.1	128.9±12.0	355.5±1.7	11.2±0.7	274.6±6.5	148.5±14.8	261.0±0.4	4.4±0.1	11.1±1.2
8-HETE	5.8±3.1	357.2±0.4	104.6±0.8	392.0±0.5	10.5±6.6	87.0±2.5	55.8±0.8	151.4±2.6	41.6±2.5	118.5±3.2
PGF_2α_	5.9±6.8	94.4±12.6	9.0±1.2	37.2±0.9	5.7±0.4	41.6±2.3	14.9±1.2	124.8±8.7	27.6±4.8	43.8±7.7
PGD_2_	6.8±1.2	324.6±1.5	BLOQ	233.2±4.0	BLOQ	234±3.9	33.7±5.2	149.6±16.1	BLOQ	72.2±5.4
PGE_2_	13.5±1.8	236.9±10.7	30.0±6.0	136.6±15.5	5.6±2.5	97.7±8.9	28.5±6.0	104.8±2.7	22.2±2.0	40.5±0.4
LTB_4_	9.4±1.2	280.0±0.2	148.1±3.4	633.5±0.3	75.8±6.1	695±2.2	157.2±18.8	848.1±0.2	134.6±0.1	347.9±0.2

Data are expressed as mean±SD. All values in tissue are expressed as ng/g except for AA in μg/g. ND = not detected, BLOQ = below level of quantification. Statistical comparisons between groups were performed using a Mann Whitney test where *p<0.05 compared to saline-treated rats

#### Prostate

The prostate is a part of the male reproductive system that wraps around the tube that carries urine out of the bladder. In BPH, smooth muscle and epithelial cells grow primarily within the prostatic transition zone, and this pathological process induces unpleasant lower urinary tract symptoms [18]. Evidence suggests that prostate tissues might produce inflammatory metabolites when BPH develops.

The level of AA and 9 metabolites detected in BPH rat prostate were significantly increased compared with levels in sham groups (P<0.05). The metabolites generated via the LOX route are highly expressed, including 15-HETE, 12-HETE, 5-HETE, 8-HETE, and LTB_4_. Levels of LTB_4_ and 8-HETE were 30-fold and 61-fold higher, respectively, compared to those in the sham group, whereas 15-HETE, 12-HETE and 5-HETE were up regulated moderately (4-6-fold). PGE_2_, PGD_2_, and PGF_2α_ are the products metabolized from linoleic acid by COX and were also highly upregulated (17, 47 and 16-fold, respectively), except for PGI_2_, which was upregulated moderately (7-fold). In this study, with prostate as the target organ, AA was detected in model rats at levels approximately 14.5 μg/g and was the highest eicosanoid detected in all of the tissues. Additionally, both LOX products (15-HETE, 8-HETE and LTB_4_) and COX products (PGI_2_, PGD_2_ and PGE_2_) were measured consistently approximately 200 ng/g. The results indicate that prostates harvested from BPH animals have a marked increase in the expression of both LOX and COX, and it lead to a greater induction of prostatic cell growth.

#### Bladder

The bladder is a hollow organ in the lower part of the abdomen that is shaped like a small balloon and has a muscular wall that allows it to become larger or smaller. It is part of the urinary tract, along with the kidneys, ureters, and urethra. The bladder stores urine until it is passed out of the body [19]. AA and 10 metabolites in BPH rat bladder tissue were significantly higher than those in bladder tissue from control rats (*P*<0.05). PGI_2_ and PGE_2_ were 24-fold and 17-fold higher compared to levels measured in sham rats, whereas 15-HETE, 12-HETE, TXA_2_, AA, PGF_2α_, 8-HETE, PGD_2_ and LTB_4_ were moderately upregulated (2-10-fold). Although the elevated levels were tissue-specific, they still maybe indicators of BPH in the bladder.

#### Seminal

AA and metabolites in seminal tissue from rats with BPH were profiled here for the first time, and the results showed a marked increase in 15-HETE, 12-HETE, TXA_2_, 5-HETE, AA, PGI_2_, PGF_2α_, 8-HETE, PGD_2_, PGE_2_ and LTB_4_ compared to levels in seminal tissue from sham group rats (*P*<0.05). Due to the positional specificity of the enzymes, LOX_S_ in the seminal tissue favored the production of 8-HETE, and its content was close to 400 ng/g and was significantly higher than in other tissues. Apart from 8-HETE, the distribution of AA metabolites in seminal tissue were similar to those in the bladder. These results suggested that the pathways generating those eicosanoids were activated during the circulation of seminal interstitial fluid during BPH development.

#### Liver

The liver tissue synthesizes various enzymes, including enzymes that are related to AA metabolism. AA and 10 metabolites were detected in BPH rat liver tissue. Most of the biomarkers (15-HETE, 12-HETE, TXA_2_, AA, PGI_2_, PGF_2α_, 8-HETE, PGD_2_, PGE_2_ and LTB_4_) were moderately upregulated (2-8-fold); the exception was 5-HETE (15-fold). It was also noteworthy that the corresponding concentration of LTB_4_ in the liver was 840 ng/g, and it was highly upregulated in BPH rats (5-fold compared to sham). PGF_2α_, one of the prostaglandins that has pro-inflammatory properties, was detected in prostates from the BPH group at levels approximately 124 ng/g, which was the highest level found among all of the tissues. A similar tendency was also found in the metabolites 15HETE, 12HETE, 5HETE and TXA_2_. A likely explanation for the high levels of AA metabolites herein is that liver activity under pathological conditions is capable of AA synthesis; once released, AA can be metabolized by COX, LOX, or CYP to produce a range of eicosanoids that can modulate the expression of ion channels, pumps, and protein kinases.

#### Kidney

Eicosanoids derived from AA are important local regulators in kidneys under inflammatory conditions. AA and metabolites regulate the glomerular filtration rate through the regulation of renal blood pressure, microvascular hemodynamics, vascular tone, and rennin release [20]. In the kidney, AA and 10 metabolites were detected, and the levels of biomarkers detected in kidney from BHP rats were significantly increased compared with sham rats (P<0.05). The greatest change was in TXA_2_ (7-fold), whereas 15-HETE, 12-HETE, 5-HETE, AA, PGI_2_, PGF_2α_, 8-HETE, PGD_2_, PGE_2_ and LTB_4_ were moderately upregulated (2-3-fold). Additionally, kidneys can express 5-, 12- and 15-LOX that produce 5-, 12-, and 15-HETE, respectively; as a consequence, all were detected with a 2-3-fold increase.

### Regional differences in eicosanoids profiles

Multivariate data analysis of BPH rat eicosanoid profile data sets using PLS-DA for five tissues emphasizes the changes in the patterns of eicosanoid distributions between tissues.

**Fig 5:**
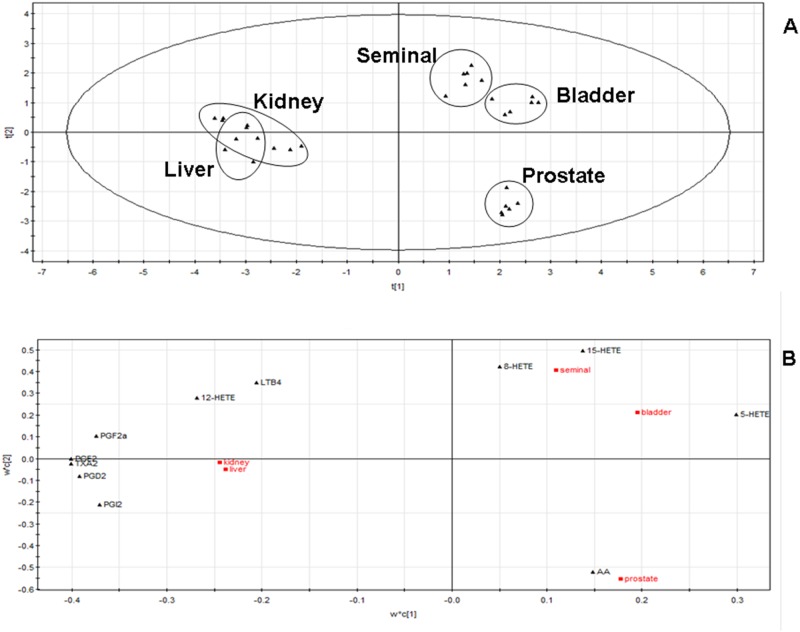
Eicosanoid profiles from each rat tissue were analyzed using PLS-DA to examine variation between samples represented by scores plot (R^2^*x*[1] = 0.446, R^2^*x*[2] = 0.183, Ellipse:Hotelling T_2_(95%)) of a two-component PLS-DA model of the dataset (A) and loadings plot of the same dataset (B).

Fig 5A showed the following three groups with similar distributions of eicosanoids: 1) prostate; 2) seminal and bladder; and 3) liver and kidney. The PLS-DA loadings plot (Fig 5B) showed the dominant eicosanoids that typify the following three groups: COX products were higher in the liver and kidney; LOX products were higher in the seminal tissue and bladder; AA was higher in the prostate and was generated via both LOX and COX.

### Profile of the tissue distribution of bioactive eicosanoids in a rat model of BPH

**Fig 6:**
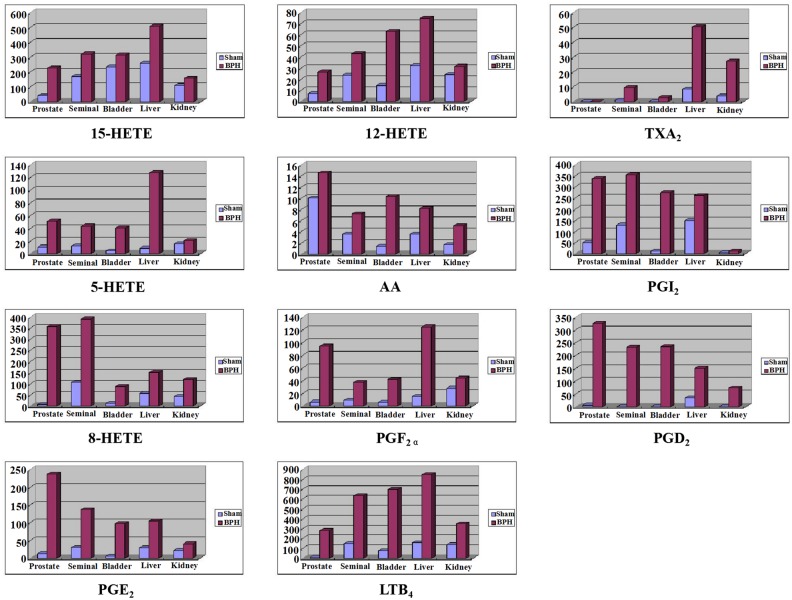
The histograms of 11 analytes in prostate, bladder, seminal, liver, kidney from BPH group rats (BPH) and sham group rats (sham).

The histograms of 11 analytes in the prostate, seminal, bladder, liver, and kidney tissues from BPH and sham rats were shown in Fig 6.

Among detectable eicosanoids metabolized from AA, LTB_4_, a powerful proinflammatory product, was measured at a content of approximately 840 ng/g; this was the highest level of AA-derived LOX products, and tissue LTB_4_ was significantly upregulated when compared to sham groups in five tissues (P<0.05). LTB_4_ attracts leukocytes to the site of inflammation, promoting their adhesion to the inflamed and damaged tissue. This result suggests that the increase in LTB_4_ may amplify the inflammatory cascade that accompanies the benign overgrowth of the prostate gland. PGE_2_ and PGD_2_, the final metabolites of the COX pathways, which are important mediators of inflammation and pain, were also significantly increased in five tissues in BPH rats. To date, there is little information on tissue levels of eicosanoids apart from this publication; only LTB_4_ and PGE_2_ in prostate tissue of rats have previously been measured using the ELISA kit/EIA kit method and that report is consistent with our results to some degree [9]. Overall, BPH fortified inducible expression of COX and LOX, as well as increased production of AA and eicosanoids. These data show that inflammation is closely associated with the development of BPH.

## Conclusions

In this study, a highly sensitive and specific UHPLC—MS/MS method was developed for the simultaneous profiling of the eicosanoid metabolome in different tissues of sham and BPH rats and ten biomarkers were found. All of these experimental findings suggest that a “dual regulator” of the COX and LOX enzymes might play a key role in the development of pathological process of BPH. The results of this study have provided valuable information about the distribution of eicosanoid lipids in different tissues in relation to their biological function and involvement in biological processes.

## Supporting Information

S1 Table List of selected MRM parameters, declustering potential (DP), entrance potential (EP), collision energy (CE), and cell exit potential (CXP) for each analytes measured. (DOC)

S2 Table Summary of regression equations, linear ranges and LLOQs of the eleven analytes in rat prostate. (DOC)
