# Supplementary material for: Quantification of Arachidonic Acid and Its Metabolites in Rat Tissues by UHPLC-MS/MS: Application for the Identification of Potential Biomarkers of Benign Prostatic Hyperplasia
Source: PLoS One. 2016 Nov 28;11(11):e0166777. doi: 10.1371/journal.pone.0166777 (PMC5125601; doi:10.1371/journal.pone.0166777)
Supplement: S1 Table — (DOC) [file pone.0166777.s001.doc]

**Table S1** List of selected MRM parameters, declustering potential (DP), entrance potential (EP), collision energy (CE), and cell exit potential (CXP) for each analytes measured.

| Name | Q1 | Q3 | DP | EP | CE | CXP |
| --- | --- | --- | --- | --- | --- | --- |
| 15-HETE | 319.2 | 301.1 | -64.1 | -11.2 | -17.6 | -8.0 |
| AA-d_8_(IS) | 311.2 | 267.2 | -69.1 | -10.0 | -18.2 | -11.4 |
| 12-HETE | 319.2 | 179.1 | -77.7 | -9.0 | -19.9 | -2.3 |
| TXA_2_ | 437.4 | 246.8 | -85.9 | -4.1 | -31.1 | -11.9 |
| PGE_2_-d_4_(IS) | 355.2 | 319.1 | -68.0 | -6.9 | -16.0 | -7.5 |
| 5-HETE | 319.2 | 114.7 | -56.8 | -7.1 | -16.2 | -6.0 |
| AA | 302.9 | 259.3 | -90.0 | -10.0 | -19.1 | -13.3 |
| PGI_2_ | 369.2 | 369.2 | -85.0 | -11.3 | -8.8 | -9.9 |
| PGF_2α_ | 353.2 | 308.9 | -59.9 | -9.1 | -25.9 | -16.0 |
| 8-HETE | 319.2 | 154.6 | -65.1 | -10.0 | -21.9 | -10.8 |
| PGD_2_ | 351.2 | 330.0 | -67.9 | -2.7 | -16.6 | -11.9 |
| PGE_2_ | 351.2 | 159.0 | -60.6 | -7.9 | -32.0 | -13.0 |
| LTB_4_ | 335.0 | 194.8 | -76.1 | -8.0 | -22.1 | -9.3 |
| 15-HETE-d_8_(IS) | 327.2 | 226.1 | -62.6 | -11.0 | -18.9 | -16.0 |

**Table S2** Summary of regression equations, linear ranges and LLOQs of the eleven analytes in rat prostate.

| Analyte | Regression equation | Regression coefficient (r) | Linear range (ng/ml) | LLOQ RSD (%) |
| --- | --- | --- | --- | --- |
| 15-HETE | *y*=0.0140*x*+0.1171 | 0.9967 | 1.25–500 | 3.9 |
| 12-HETE | *y*=0.2655*x*－0.2817 | 0.9958 | 0.25–100 | 5.1 |
| TXA_2_ | *y*=0.0395*x*+0.0844 | 0.9961 | 0.625–250 | 8.2 |
| 5-HETE | *y*=0.1247*x*－0.0945 | 0.9989 | 1.25–500 | 0.8 |
| AA | *y*=0.0017*x*+0.5850 | 0.9977 | 50–20 000 | 9.5 |
| PGI_2_ | *y*=0.0425*x*+0.1658 | 0.9974 | 1–400 | 7.2 |
| PGF_2α_ | *y*=0.0091*x*+0.0829 | 0.9977 | 1.25–500 | 1.5 |
| 8-HETE | *y*=0.0208*x*+0.0557 | 0.9955 | 0.625–250 | 3.7 |
| PGD_2_ | *y*=0.0194*x*+0.4636 | 0.9943 | 1.25–500 | 6.9 |
| PGE_2_ | *y*=0.0211*x*+0.0451 | 0.9964 | 1–400 | 5.7 |
| LTB_4_ | *y*=0.0024*x*+0.0621 | 0.9960 | 1.25–500 | 6.1 |
