# Supplementary material for: Quantification of Arachidonic Acid and Its Metabolites in Rat Tissues by UHPLC-MS/MS: Application for the Identification of Potential Biomarkers of Benign Prostatic Hyperplasia
Source: PLoS One. 2016 Nov 28;11(11):e0166777. doi: 10.1371/journal.pone.0166777 (PMC5125601; doi:10.1371/journal.pone.0166777)
Supplement: S2 Table — (DOC) [file pone.0166777.s002.doc]

| bladder |  |  |  |  |  |  |  |  |  |  |  |
| --- | --- | --- | --- | --- | --- | --- | --- | --- | --- | --- | --- |
|  | 15-HETE | 12-HETE | TXA2 | 5-HETE | AA | PGI2 | 8-HETE | PGF2a | PGD2 | PGE2 | LTB4 |
| BPH | 4.356436 | 19.14191 | 0.706271 | 6.732673 | 10.29703 | 10.62706 | 0.732673 | 1.10231 | 2.759076 | 0.90429 | 1.768977 |
| BPH | 5.141243 | 13.75706 | 1.666667 | 11.46893 | 10.56497 | 7.118644 | 1.016949 | 0.084746 | 2.711864 | 1.954802 | 1.519774 |
| BPH | 5.654762 | 12.2619 | 2.494048 | 18.21429 | 10.41667 | 6.071429 | 1.64881 | 1.011905 | 2.35119 | 1.85119 | 1.363095 |
| BPH | 4.358354 | 11.64649 | 1.031477 | 9.443099 | 9.249395 | 7.893462 | 0.692494 | 0.716707 | 2.256659 | 1.549637 | 1.191283 |
| BPH | 4.913793 | 9.482759 | 1.284483 | 9.008621 | 8.362069 | 4.956897 | 0.818966 | 6.952586 | 1.969828 | 1.267241 | 1.237069 |
| BPH | 4.652956 | 11.491 | 1.305913 | 8.226221 | 10.53985 | 8.688946 | 1.095116 | 0.848329 | 2.344473 | 1.59383 | 1.434447 |
| Sham | 1.832669 | 4.103586 | 0.101195 | 0.741036 | 3.705179 | 4.621514 | 0.36255 | 0.283267 | 0.705179 | 0.401195 | 0.191235 |
| Sham | 2.637363 | 5.641026 | 0.117949 | 0.622711 | 6.080586 | 5.567766 | 0.512821 | 0.32381 | 0.396337 | 0.220513 | 0.194872 |
| Sham | 2.885375 | 6.561265 | 0.255375 | 0.50751 | 6.007905 | 4.84585 | 0.537549 | 0.373913 | 0.675889 | 0.703557 | 0.310672 |
| Sham | 2.816092 | 6.695402 | 0.561494 | 0.885057 | 4.310345 | 4.12069 | 0.425287 | 0.228161 | 1.362069 | 0.58046 | 0.448276 |
| Sham | 1.977273 | 4.431818 | 0.463636 | 0.404545 | 3.454545 | 3.495455 | 0.304545 | 0.160909 | 1.109091 | 0.354545 | 0.322727 |
| Sham | 2.994652 | 4.86631 | 0.342513 | 0.44385 | 3.68984 | 3.593583 | 0.385027 | 0.2 | 1.144385 | 0.411765 | 0.413904 |
|  |  |  |  |  |  |  |  |  |  |  |  |
| seminal |  |  |  |  |  |  |  |  |  |  |  |
|  | 15-HETE | 12-HETE | TXA2 | 5-HETE | AA | PGI2 | 8-HETE | PGF2a | PGD2 | PGE2 | LTB4 |
| BPH | 3.228448 | 6.810345 | 0.377586 | 4.594828 | 9.310345 | 12.24138 | 2.952586 | 0.67069 | 4.258621 | 1.954023 | 1.146552 |
| BPH | 3.279352 | 8.906883 | 0.406478 | 3.287449 | 9.230769 | 11.94332 | 2.611336 | 0.651822 | 3.910931 | 2.024291 | 1.587045 |
| BPH | 3.05618 | 7.827715 | 0.331086 | 3.101124 | 9.363296 | 8.876404 | 2.078652 | 0.557303 | 3.340824 | 2.347066 | 1.250936 |
| BPH | 3.455882 | 7.132353 | 0.333824 | 4.338235 | 8.676471 | 10.55147 | 2.959559 | 0.553676 | 3.514706 | 2.389706 | 0.926471 |
| BPH | 2.633333 | 7.266667 | 0.290667 | 3.146667 | 8.4 | 9.766667 | 2.45 | 0.508 | 3.073333 | 1.5 | 0.78 |
| BPH | 3.060897 | 6.153846 | 0.284615 | 2.5 | 7.5 | 8.044872 | 1.714744 | 0.487179 | 2.782051 | 1.581197 | 0.762821 |
| Sham | 0.941176 | 4.173669 | 0.084594 | 0.812325 | 4.089636 | 3.557423 | 0.533053 | 0.231933 | 0.394958 | 0.455649 | 0.403361 |
| Sham | 1.584158 | 4.009901 | 0.075248 | 0.871287 | 3.366337 | 2.871287 | 0.60396 | 0.15198 | 0.297525 | 0.567657 | 0.277228 |
| Sham | 1.658768 | 3.459716 | 0.100474 | 0.616114 | 4.597156 | 4.075829 | 0.625592 | 0.112796 | 0.287204 | 0.383886 | 0.279621 |
| Sham | 1.935897 | 3.621795 | 0.122436 | 1.134615 | 4.679487 | 3.685897 | 0.918269 | 0.130128 | 0.402564 | 0.526709 | 0.307692 |
| Sham | 2.021978 | 3.846154 | 0.156777 | 0.981685 | 6.007326 | 4.139194 | 1 | 0.220513 | 0.545055 | 0.40293 | 0.271062 |
| Sham | 2.092199 | 4.255319 | 0.104965 | 1.255319 | 4.609929 | 4.574468 | 0.623404 | 0.236879 | 0.524823 | 0.460993 | 0.255319 |
|  |  |  |  |  |  |  |  |  |  |  |  |
| liver |  |  |  |  |  |  |  |  |  |  |  |
|  | 15-HETE | 12-HETE | TXA2 | 5-HETE | AA | PGI2 | 8-HETE | PGF2a | PGD2 | PGE2 | LTB4 |
| BPH | 4.407407 | 12.81481 | 0.177963 | 5.481481 | 14.7037 | 9.333333 | 0.637037 | 0.775926 | 2.75 | 2.244444 | 0.855556 |
| BPH | 2.923077 | 9.480769 | 0.162308 | 0.451923 | 15.30769 | 6.653846 | 0.617308 | 0.696154 | 3.548077 | 0.848077 | 1.355769 |
| BPH | 2.698113 | 12.50943 | 0.159057 | 3.584906 | 11.13208 | 10.11321 | 0.579245 | 0.577358 | 3.118868 | 1.454717 | 1.009434 |
| BPH | 2.788462 | 7.538462 | 0.110962 | 2.673077 | 10.25 | 7.826923 | 0.582692 | 0.509615 | 3.634615 | 1.678846 | 1.317308 |
| BPH | 2.823529 | 10.84314 | 0.185294 | 4.45098 | 12.80392 | 4.313725 | 0.621569 | 0.713725 | 3.582353 | 0.835294 | 1.329412 |
| BPH | 2.627451 | 12 | 0.115686 | 3.039216 | 9.058824 | 9.431373 | 0.627451 | 0.535294 | 4.347059 | 1.458824 | 1.184314 |
| Sham | 1.962775 | 1.128596 | 0.066159 | 0.22335 | 1.184433 | 0.651438 | 0.142132 | 0.080034 | 0.416244 | 0.153976 | 0.230118 |
| Sham | 1.597276 | 2.081712 | 0.039105 | 0.309339 | 1.906615 | 0.354086 | 0.123152 | 0.108171 | 0.51751 | 0.124514 | 0.243191 |
| Sham | 2.719735 | 2.752902 | 0.040464 | 0.154229 | 2.155887 | 0.45937 | 0.128192 | 0.112106 | 0.427861 | 0.07131 | 0.128027 |
| Sham | 2.5 | 1.381356 | 0.042373 | 0.216102 | 2.754237 | 0.415254 | 0.173729 | 0.132203 | 0.597458 | 0.188559 | 0.17161 |
| Sham | 2.661738 | 1.848429 | 0.037893 | 0.297597 | 2.218115 | 0.365989 | 0.173937 | 0.115712 | 0.417745 | 0.125693 | 0.136229 |
| Sham | 2.368421 | 4.385965 | 0.05386 | 0.257895 | 2.631579 | 0.650877 | 0.139825 | 0.259649 | 0.382456 | 0.078947 | 0.191228 |
|  |  |  |  |  |  |  |  |  |  |  |  |
| kidney |  |  |  |  |  |  |  |  |  |  |  |
|  | 15-HETE | 12-HETE | TXA2 | 5-HETE | AA | PGI2 | 8-HETE | PGF2a | PGD2 | PGE2 | LTB4 |
| BPH | 4.407407 | 12.81481 | 1.77963 | 5.481481 | 14.7037 | 0.933333 | 1.592593 | 1.551852 | 1.833333 | 1.87037 | 0.855556 |
| BPH | 2.923077 | 9.480769 | 1.623077 | 0.451923 | 15.30769 | 0.665385 | 1.543269 | 1.392308 | 2.365385 | 0.706731 | 1.355769 |
| BPH | 2.698113 | 12.50943 | 1.590566 | 3.584906 | 11.13208 | 1.011321 | 1.448113 | 1.154717 | 2.079245 | 1.212264 | 1.009434 |
| BPH | 2.788462 | 7.538462 | 1.109615 | 2.673077 | 10.25 | 0.782692 | 1.456731 | 1.019231 | 2.423077 | 1.399038 | 1.317308 |
| BPH | 2.823529 | 10.84314 | 1.852941 | 4.45098 | 12.80392 | 0.431373 | 1.553922 | 1.427451 | 2.388235 | 0.696078 | 1.329412 |
| BPH | 2.627451 | 12 | 1.156863 | 3.039216 | 9.058824 | 0.943137 | 1.568627 | 1.070588 | 2.898039 | 1.215686 | 1.184314 |
| Sham | 1.103448 | 2.068966 | 0.164138 | 1.068966 | 1.586207 | 0.25 | 0.298276 | 0.193103 | 0.5 | 0.305603 | 0.25 |
| Sham | 1.627451 | 1.568627 | 0.148039 | 1.098039 | 3.235294 | 0.343137 | 0.307843 | 0.482353 | 0.52549 | 0.436275 | 0.148824 |
| Sham | 0.341463 | 4.439024 | 0.203415 | 1.317073 | 4.658537 | 0.37561 | 0.343902 | 0.453659 | 0.485854 | 0.493902 | 0.414634 |
| Sham | 1.824074 | 4.944444 | 0.13963 | 1.537037 | 2.277778 | 0.167037 | 0.27037 | 0.32963 | 0.297037 | 0.293056 | 0.152778 |
| Sham | 1.208333 | 6.854167 | 0.1625 | 1.208333 | 1.604167 | 0.297917 | 0.370833 | 0.545833 | 0.38375 | 0.333333 | 0.200833 |
| Sham | 0.686275 | 3.960784 | 0.282353 | 0.843137 | 1.490196 | 0.303922 | 0.455882 | 0.627451 | 0.36 | 0.284314 | 0.498039 |
|  |  |  |  |  |  |  |  |  |  |  |  |
| prostate |  |  |  |  |  |  |  |  |  |  |  |
|  | 15-HETE | 12-HETE | TXA2 | 5-HETE | AA | PGI2 | 8-HETE | PGF2a | PGD2 | PGE2 | LTB4 |
| BPH | 2.219388 | 6.810345 | 0.377586 | 4.594828 | 9.310345 | 12.24138 | 2.952586 | 0.67069 | 4.258621 | 1.954023 | 1.146552 |
| BPH | 2.14486 | 8.906883 | 0.406478 | 3.287449 | 9.230769 | 11.94332 | 2.611336 | 0.651822 | 3.910931 | 2.024291 | 1.587045 |
| BPH | 2.289216 | 7.827715 | 0.331086 | 3.101124 | 9.363296 | 8.876404 | 2.078652 | 0.557303 | 3.340824 | 2.347066 | 1.250936 |
| BPH | 2.388601 | 7.132353 | 0.333824 | 4.338235 | 8.676471 | 10.55147 | 2.959559 | 0.553676 | 3.514706 | 2.389706 | 0.926471 |
| BPH | 1.990698 | 7.266667 | 0.290667 | 3.146667 | 8.4 | 9.766667 | 2.45 | 0.508 | 3.073333 | 1.5 | 0.78 |
| BPH | 2.411168 | 6.153846 | 0.284615 | 2.5 | 7.5 | 8.044872 | 1.714744 | 0.487179 | 2.782051 | 1.581197 | 0.762821 |
| Sham | 0.941176 | 4.173669 | 0.084594 | 0.812325 | 4.089636 | 3.557423 | 0.533053 | 0.231933 | 0.394958 | 0.455649 | 0.403361 |
| Sham | 1.584158 | 4.009901 | 0.075248 | 0.871287 | 3.366337 | 2.871287 | 0.60396 | 0.15198 | 0.297525 | 0.567657 | 0.277228 |
| Sham | 1.658768 | 3.459716 | 0.100474 | 0.616114 | 4.597156 | 4.075829 | 0.625592 | 0.112796 | 0.287204 | 0.383886 | 0.279621 |
| Sham | 1.935897 | 3.621795 | 0.122436 | 1.134615 | 4.679487 | 3.685897 | 0.918269 | 0.130128 | 0.402564 | 0.526709 | 0.307692 |
| Sham | 2.021978 | 3.846154 | 0.156777 | 0.981685 | 6.007326 | 4.139194 | 1 | 0.220513 | 0.545055 | 0.40293 | 0.271062 |
| Sham | 2.092199 | 4.255319 | 0.104965 | 1.255319 | 4.609929 | 4.574468 | 0.623404 | 0.236879 | 0.524823 | 0.460993 | 0.255319 |
